# Role of Ascorbic acid, Glutathione and Proline Applied as Singly or in Sequence Combination in Improving Chickpea Plant through Physiological Change and Antioxidant Defense under Different Levels of Irrigation Intervals

**DOI:** 10.3390/molecules25071702

**Published:** 2020-04-08

**Authors:** Hossam S. El-Beltagi, Heba I. Mohamed, Mahmoud R. Sofy

**Affiliations:** 1 Agricultural Biotechnology Department, College of Agriculture and Food Sciences, King Faisal University, P.O. Box 420, Al-Ahsa 31982, Saudi Arabia; 2 Biochemistry Department, Faculty of Agriculture, Cairo University, Gamma St., P.O. Box 12613 Giza, Cairo, Egypt; 3 Biological and Geological Sciences Department, Faculty of Education, Ain Shams University, Roxy, P.C.11757 Heliopolis, Cairo, Egypt; 4 Botany and Microbiology Department, Faculty of Science, Al-Azhar University, 11884 Nasr City, Cairo, Egypt

**Keywords:** antioxidant, yield, enzymatic and nonenzymatic antioxidants, protein, photosynthetic pigments

## Abstract

In recent years, the harmful effects of drought stress have been be mitigated by using bioactive compounds such as antioxidants and osmolytes. In this research, pot experiments were carried out to investigate the effects of ascorbic acid, glutathione and proline on alleviating the harmful effect of drought stress in chickpea plants during season 2017. Chickpea plant seeds were soaked in ascorbic acid (0.75 mM), glutathione (0.75 mM), proline (0.75 mM) singly and/or in sequence combinations for 4 h and then planted in pots. The pots were irrigated with water after seven days (to serve as control), after 14 days (moderate drought stress) and after 28 days (severe drought stress). The sequence combination of antioxidants and proline under drought stress has not been studied yet. The results showed significantly decreased in plant growth, yielding characteristics, photosynthetic pigments and soluble protein content in response to moderate and severe drought stress. Moreover, treatment with antioxidants caused increment the antioxidant enzyme activity, non-enzymatic antioxidant (ascorbic acid and glutathione) contents and endogenous proline in stressed and unstressed plants. In conclusion, The sequence combination of antioxidants and proline caused improvement in plant growth under drought stress by up-regulating the antioxidant defense system and osmolyte synthesis.

## 1. Introduction

All over the world, especially in arid and semi-arid regions that were suffering from water limitation, which is considered the most harmful stresses in the plant environment and reduces plant growth and development [1]. Prolongation of the duration of the water stress causes significant damage and consequently plant cell death due to the generation of excess reactive oxygen species (ROS) which causes lipid peroxidation, degradation of proteins and nucleic acids causing an alteration in plant metabolism [2,3]. About 45% of world agricultural land suffers from drought stress [4]. Increasing plant resistance to drought stress would be the most economical approach to improving agricultural productivity and reducing agricultural use of freshwater resources [5]. To cope ROS effects, plants generate antioxidants that include both enzymatic and non-enzymatic components, phenolic compounds and several phytohormones that regulate the mechanisms to maintain optimum ROS concentration [6,7]. In addition, general metabolic adaptation, which enables plants to cope with water or osmotic stress, involves an increased synthesis of osmoprotectants, such as proline (Pro) [8].

Due to a worldwide decline in water available for the agricultural sector, for causes such as a rapidly growing population, significant impacts of climate change, global warming and several human activities [9]. Some strategies need to be used including osmolytes and antioxidants, to support plant growth under stress such as proline [10], ascorbic acid [11] and glutathione [12]. Ascorbic acid (AsA) acts as the transport of antioxidants and electrons [12]; as an enzyme co-factor and retains physiological and signaling pathways regulated by phytohormones [11], neutralizes ROS directly through the use of secondary antioxidants during the reduction of the oxidized form of α-tocopherol [13] and is a significant plant metabolite and acts as a cell signaling modulator in many physiological processes such as mitosis [14]. In addition, treatment with AsA increased the growth of quinoa plants and alleviates the harmful effects of drought stress [15].

Glutathione (GSH) is a water-soluble thiol compound, low molecular weight, widely distributed in most plant tissues and participates directly or indirectly in detoxification of ROS [16]. Moreover, GSH help in the sequester of heavy metals in the vacuoles by formation of phytochelatins (PCs) [17], Cell proliferation, growth and development are modulated by Glutathione [18]. Exogenous GSH may enhance abiotic stress plant tolerances [19].

Proline (Pro) is an osmoprotectant and primarily produced in relative amounts in plants under abiotic stress [4]. Many functions are suggested for proline accumulation in plant tissues subjected to abiotic stress, such as drought stress [5], protein and membrane stabilization, osmoprotectant and free radical scavenging [20,21]. The level of free proline is regulated by the ratio of the rates of its biosynthesis and degradation [22]. Proline has been shown to function as a molecular chaperone able to protect protein integrity and enhance the activities of different enzymes [23]. Several studies have, therefore, shown that exogenous proline applications can alleviate the harmful effect of environmental stresses such as drought [4]. Chickpea (*Cicer arietinum* L.) is acritical crop of pulses that have been grown and consumed throughout the world, particularly in African and Asian countries. It contains high amounts of carbohydrates, protein, and substantial amounts of essential amino acids except sulfur-containing types that can be complemented by adding cereals to the daily diet [24]. It can be a beneficial legume crop for short-term rotation incorporation and nitrogen fixation in soil and fertility [25].

As far as we knew, there are many reports about assessing the effects of exogenous antioxidants applied as individual treatments on various plant species growing under abiotic stress conditions, however no previous reports are conducted about the effects of exogenous antioxidants and proline applied as sequenced treatments on plants under drought stress until now but there are reports about other abiotic stress such as cadmium. Therefore, the objective of the present study is to investigate the impact of exogenous AsA, GSH and Pro applied singly or in a sequence of growth, photosynthetic pigment, proline, non-enzymatic and enzymatic antioxidant defense systems under drought stress.

## 2. Results and Discussion

### 2.1. Changes in Morphological Characteristics

Data in Table 1 show that shoot and root lengths, fresh and dry weight of shoots and roots of chickpea plants were significantly decreased under different irrigation interval (14 and 28 days) as compared with normal plants irrigated at 7 days. The irrigation after 14 and 28 days was considerate as moderate and severe drought stress respectively. Treatment with ascorbic acid, glutathione, proline and combination caused significantly increased in all growth characteristics in non-stressed and stressed plants as compared with non- stressed plants. Proline was considered to be the most effective treatment after combination with AsA, GSH and Prol in alleviating drought stress followed by GSH.

### 2.2. Changes in Yield Attribute

In comparison with well-watered plants, yield attributes (No of pods/plant, weight of pods/plant, No of seeds/plant and weight of 100 seeds) were significantly reduced under moderate and severe drought stress (14- and 28-day irrigation intervals) in chickpea plants (Table 2). However, AsA, GSH and Pro treatments caused stimulation in all the above criteria as compared with untreated plants. In addition, the combination between treatments gives the highest increment as compared with each treatment alone and control plants.

Drought stress in arid and semi-arid climates that suffer from limited water supply to roots, the rapid rise in transpiration rates and higher temperatures can affect plant growth [26].

Our results showed that moderate and severe drought stress caused a reduction in chickpea growth and yield. Decreases in the growth of common bean plants [27], wheat plant [28] and soybean plants [29,30,31] have been recorded as a result of drought stress. A decline in plant growth due to drought stress may be attributed to the deregulation of elongating cells due to disruption of the flow of water from the xylem to elongating cells, reduction in the growth-promoting hormones, cell elongation, cell expansion and mitosis during cell division [32]. Global, climate change causes drought stress events [2]. Therefore, new management will be required to deal with this problem by using antioxidants and or osmoprotectant. Treatment of chickpea plants with AsA, GSH, Pro and combination caused stimulation in plant growth and yield. Several studies have shown that foliar application of AsA in various crops such as cucumber can reduce the drastic effects of drought stress on plant growth due to the up-regulation of antioxidant system and osmoprotectant metabolism [33]. Also, exogenous application of proline modulates drought stress by stimulating the plant growth, which accomplished by inducing the antioxidant mechanism, alleviating oxidative damage, improving compatible solutes synthesis and accelerating proline accumulation, reflecting the improvement of photosynthesis and yield attributes [34]. Furthermore, the application of exogenous GSH developed growth of *Arabidopsis thaliana* under drought stress [19] due to accumulation of proline, which adjust osmotic potential and could help to maintain plant water content that is important in the plant growth and development [35].

### 2.3. Changes in Photosynthetic Pigments

**Figure 1:**
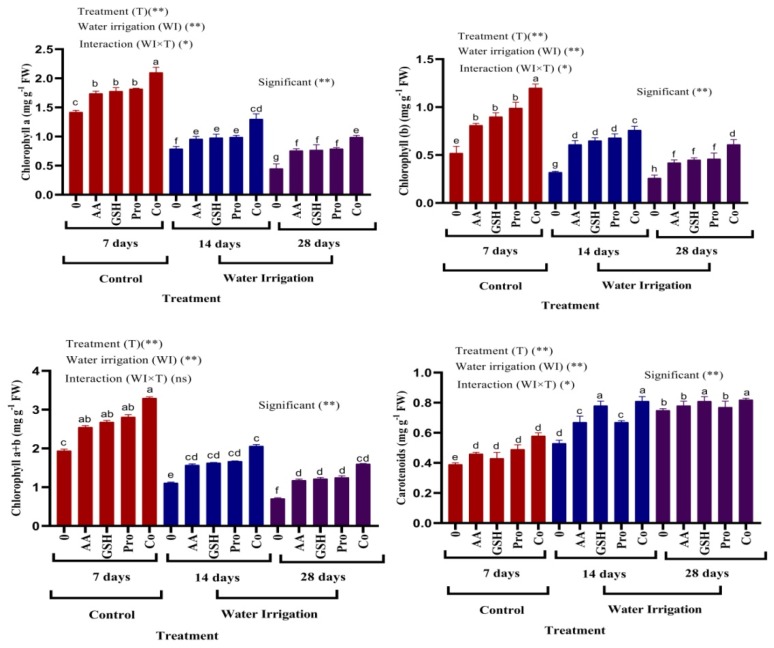
Effect of ascorbic acid, glutathione, and proline on photosynthetic pigments in leaves of chickpea plants under different irrigation intervals. a, b, c, d, e, f, g, h: Different letters next to the mean values in each column indicate significant difference (LSD *p* ≤ 0.05). *and ** indicate different at *p* value <0.05, 0.01 probability level respectively, “ns” indicate non-significant.

The results illustrated in Figure 1 show degradation in chlorophyll a, b and chlorophyll a + b content in chickpea leaves with increasing days of irrigation intervals (14 and 28 days) as compared with unstressed plants (7-day irrigation). On the other hand, carotenoid content showed a sharp increase under drought stress. Treatment with AsA, GSH and Pro showed significantly increased in chlorophyll a, b, chlorophyll a+ b and carotenoid content comparing with stressed plants. The most pronounced increases were detected in plants treated with a combination of all treatments followed by proline.

Exposure of the chickpea plant to moderate and severe drought stress caused oxidative stress, which reduced chlorophyll content, including chl a, chl b and carotenoid content. This reduction may be due to the pigment photo-oxidation and the degradation of chlorophyll. Similar results are reported in cotton and soybean plants [30,31]. Plants respond to drought stress by closing stomata to limit water loss, reducing carbon flow [36] and reducing ATP synthase and Rubisco activities [37]. Treatment with antioxidants and proline caused enhancement in the photosynthetic pigments in chickpea plants. Down-regulated chlorophyllase activity and up-regulated expression of chlorophyll biosynthetic genes may result in increased pigment synthesis by the application of antioxidants [33]. AsA play a vital role in photosynthesis and proline synthesis [38]. Also, AsA can quench ROS resulted from water stress and help in the maintenance of chlorophyll content which stimulates the level of plant growth [39]. Exogenous proline can interact with enzymes to preserve protein structures and activities [40] and play a critical role in protecting photosynthetic activity [41]. Under oxidative stress, GSH can prevent chlorophyll degradation and can protect enzymes responsible for the biosynthesis of chlorophyll which increased chlorophyll content [42].

### 2.4. Changes in Proline, Protein and non-Enzymatic Antioxidants

**Figure 2:**
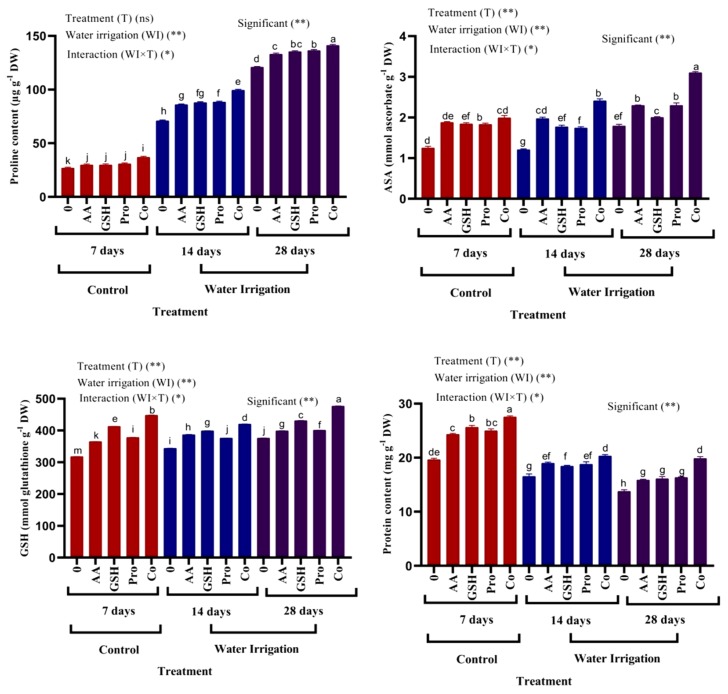
Effect of ascorbic acid, glutathione and proline on proline, non-enzymatic antioxidants and protein content in shoots of chickpea plants under different irrigation intervals. a, b, c, d, e, f, g, h: Different letters next to the mean values in each column indicate significant difference (LSD p ≤ 0.05). *and ** indicate different at *p* value <0.05, 0.01 probability level respectively, “ns” indicate non-significant.

Proline content in shoots of chickpea plants significantly increased under moderate and severe drought stress as compared with untreated plants (Figure 2). Treatment with AsA, GSH and Pro showed an insignificant effect in proline content as compared with a control plant which irrigated after 7 days, but the combination between treatments showed significant increases in endogenous proline content. Moreover, under moderate and severe drought stress, proline content significantly increased in plants treated with AsA, GSH, Pro and combination as compared with untreated plants.

The data in Figure 2 show that protein content in shoots of chickpea plants significantly decreased under moderate and severe drought stress. In addition, treatment with AsA, GSH, Pro and combination helped the plants alleviate the adverse effect of drought by increasing protein content as compared with untreated plants. The combination of treatments was the most effective one. Endogenous antioxidants (AsA and GSH) significantly increased under moderate and severe drought stress as compared with control plants. Moreover, the application of AsA, GSH, Pro and combination alleviated the moderate and severe drought stress by an induced the accumulation of non-enzymatic antioxidants.

Exogenous application of AsA, GSH, Pro and combination between all treatments showed significantly increased in endogenous AsA, GSH and Pro under moderate and severe drought stress. Exogenous application of proline increased endogenous proline content which lowers cell osmotic potential and helps in water intake; resulting in increased plant growth [43]. Foliar application of AsA will increase intrinsic quality of AsA under abiotic stress such as drought [44]. Proline played a protective role in oxidative stress caused by drought by reduction of H_2_O_2_ levels and increment of the antioxidant defense system [45]. Moreover, under drought stress, treatment with GSH increased the endogenous AsA and GSH levels in mung bean seedlings [18], which could be a preventive mechanism since a higher level of GSH has been shown to effectively stop free radicals and reduce oxidative damage [46]. GSH plays a crucial role in regeneration of water-soluble antioxidant AsA through ASC–GSH cycle [47], which could also help to increase level of AsA under drought stress. Protein content decreased under moderate and severe drought in chickpea plants. Treatment of antioxidants and proline modulates the adverse effect of drought. Proteins are molecules of crucial importance for all cell functions [48]. Water stress caused the reduction in protein synthesis, which may be due to the decrease in the number of polysomal complexes in tissues with lower water content, thus causing a reduction in plant growth and crop yield [49]. It seems that a severe decrease in photosynthesis has resulted in a decrease in soluble protein during drought stress. Therefore, photosynthesis decreased in drought stress, reducing or even stopping protein synthesis [50] Proteolysis caused progressive reduction of total soluble proteins during plant water deficiency with released amino acids used during the plant osmotic adjustment; this fact suggests a slow recovery of this parameter possibly because proteins are dependent on synthesis of other nitrogen compounds [51]. Our results were in agreement with Bayramov et al. [52]. Exogenous proline application protects plant proteins by binding to protein surfaces, thus stabilizing the native protein structure and membranes from damage caused by excess ROS levels [53]. Proline has also been shown to function as a chemical chaperone protein and to prevent the aggregation and thermal denaturation of proteins [54].

### 2.5. Changes in Enzymatic Antioxidants

**Figure 3:**
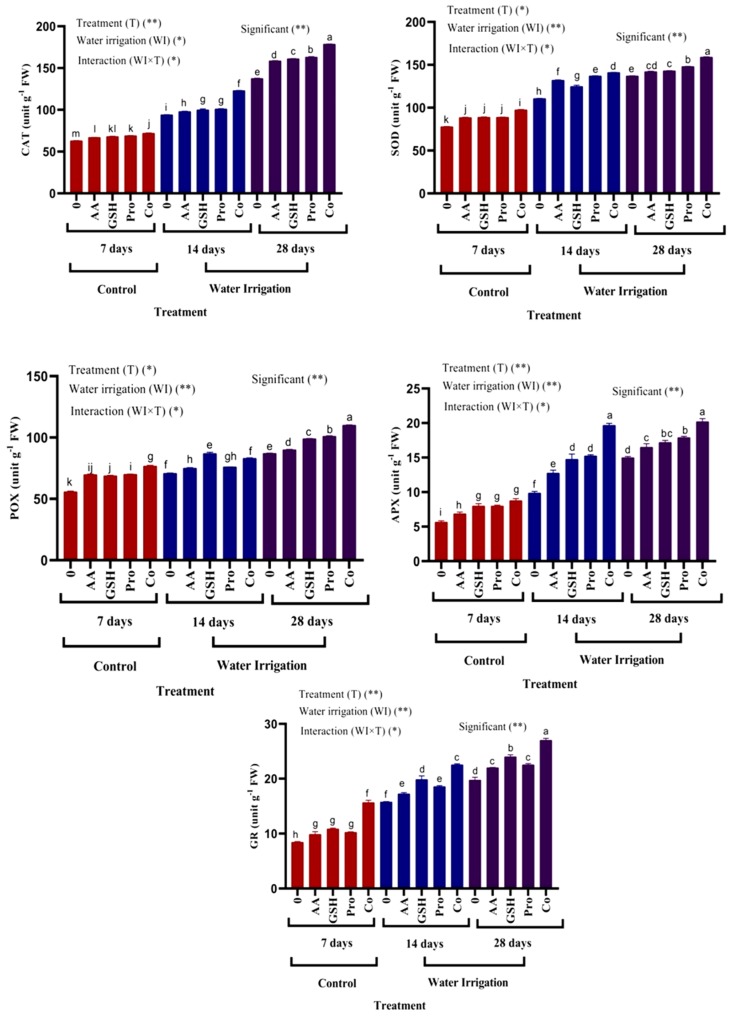
Effect of ascorbic acid, glutathione and proline on the activity of antioxidant enzymes in shoots of chickpea plants under different irrigation intervals. a, b, c, d, e, f, g, h: Different letters next to the mean values in each column indicate significant difference (LSD *p* ≤ 0.05). *and ** indicate different at *p* value <0.05, 0.01 probability level respectively, “ns” indicate non-significant.

The activities of antioxidant enzymes (CAT, SOD, POX, APX and GR) were markedly enhanced in shoots of chickpea plants under moderate and severe drought stress (Figure 3). There were insignificant differences in CAT and SOD content in plants treated with AsA, GSH, Pro and in APX and GR in plants treated with AsA, as compared with control plants. However, the combination between treatments caused significantly increased in the antioxidant enzymes as compared with control plants (7 days irrigation). Moreover, all treatments help the plant to alleviate the moderate and severe drought stress by accumulation of the antioxidant enzymes as compared with untreated plants. The sequence combination between treatments was the most effective treatment.

Activities of antioxidant enzymes (CAT, SOD, POX, APX and GR) were markedly enhanced in shoots of chickpea plants under moderate and severe drought stress. To cope with ROS, plants provides enzymatic (SOD, CAT, GPX and APX) and non-enzymatic antioxidant defense that helps to reduce oxidative damage and improve plant tolerance and resistance to drought and salt stress [55,56].Treatment with antioxidants, especially the combined antioxidants and proline led to synergistic effects between them and caused enhancement in enzymatic antioxidants in chickpea plants. Similar results have been reported by Medeiros et al. [57], who found that there was an increase in APX activity in sugarcane using 20 mM proline. Furthermore, ASA foliar application increased CAT, SOD and Calendula POX activity [58]. Increased CAT, SOD and POX activity could effectively detoxify harmful ROS effects [59]. SOD is the first ROS scavenging enzyme that converses the H_2_O_2_ to O_2_ and H_2_O [60]. Also, CAT and APX changed the switching of H_2_O_2_, a dominant and detrimental oxidizing factor to H_2_O and O_2_ [61]. De Freitas et al. [62] found that treatment with proline caused significant differences in APX activity in maize plants under salt stress. GR activity increased after exogenous GSH application; thus, it may be involved in recycling and increasing endogenous GSH [63]. Moreover, exogenous GSH enhanced GR activity in shoots of chickpea plants. This indicates that GSH, itself can stabilize the structure of biologic macromolecules and protect the enzyme and structural protein groups of sulfhydryl (-SH) against oxidation [64]. Furthermore, the high GSH content promoted the AsA–GSH cycle, thereby increasing the AsA content and raising activities of APX and GR. The combined effects of these enzymes result in removal of ROS [65]. Previous studies have reported an increase in GSH content of drought and heat-resistant plants due to an increase in GSH synthesis and/or GSH degradation [66]. Exogenous GSH has been more effective than AsA in promoting SOD, APX and GR activities with higher levels of AsA and GSH, suggesting that exogenous GSH stimulates the AsA–GSH cycle more effectively [67,68]. 

**Figure 4:**
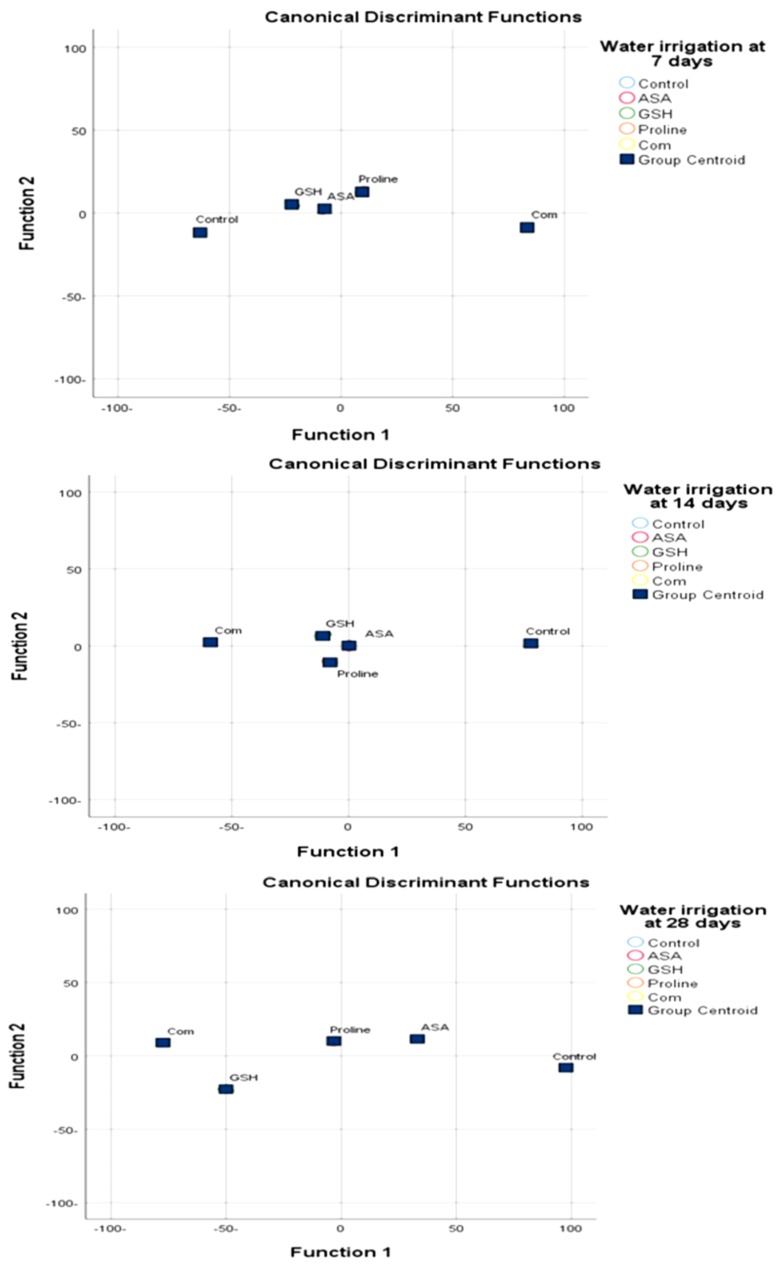
Discriminant functions show the response of AsA, GSH, Pro and sequence combination and different levels of irrigation interval in chickpea plant.

Proline was the most effective treatment after combination between all treatments under drought stress because when exposed to stressful conditions, plants accumulate an array of metabolites, particularly amino acids. Amino acids have traditionally been considered as precursors to and constituents of proteins and play an important role in plant metabolism and development [53]. A large body of data suggests a positive correlation between proline accumulation and plant stress. The most effective treatments after combination treatments were proline (Figure 4). Proline is an amino acid that plays a highly beneficial role in plants exposed to various stress conditions. Besides acting as an excellent osmolyte, proline plays three major roles during stress, i.e., as a metal chelator, an antioxidative defense molecule and a signaling molecule [53]. Proline functions not only as a compatible osmolyte but also as a radical scavenger. Thus, proline exhibited a dual function as an osmolyte compound and as an antioxidant [5]. The accumulation of proline in plants is responsible to the accumulation in the human body. In addition, the tripeptide glycine–proline–glutamate was an essential neuromodulator and neuroprotective agent in the Central nervous system (CNS). It protects neurons from diverse induced brain injuries, and rescue cell mortality in numerous models of neurodegenerative diseases, such as Parkinson’s, Alzheimer’s and Huntington’s [69,70].

### 2.6. Discriminate Analysis

Depending on the discriminate analysis, proline was considered to be the most effective treatment after combination with AsA, GSH and Pro in alleviating drought stress followed by GSH (Figure 4).

## 3. Materials and Methods

### 3.1. Plant Material

Seeds of chickpea plant (*Cicer arietinum* L.) (Var. Sena-1) were obtained from Legume Research Department, Field Crops Institute, Agriculture Research Center, Giza, Egypt.

### 3.2. Experimental Procedures

The experiment was carried out on Botanical farm, Faculty of Science, Al-Azhar University, Cairo, Egypt during winter season (2017). A solution of 0.1% mercury chloride is used to sterilize homogeneous seeds for 2 min, washed in distilled water at 25 °C three times, and then left to dry for 1 h. The seeds were soaked in ascorbic acid (AsA) (0.75 mM), glutathione (GSH) (0.75 mM), proline (Pro) (0.75 mM) for 4 h and in sequence combined; first in AsA (0.75 mM) for 90 min, then in GSH (0.75 mM) for 80 min, and finally in Pro (0.75 mM) for 70 min. Five homogenous seeds were sown in each pot (30 cm in diameter) which containing 8.0 Kg of clay soil. The soil characteristics are as follows: texture sandy loam, 25.22% sand; 21.61% silt; 45.6% clay. The chemical analyses were estimated in the soil samples taken at a depth of 30 cm before planting according to Nelson and Sommers [71] method and described in Table 3.

Pots were arranged in a completely randomized block design which divided into three groups representing the following treatments, each treatment contains six replications and The different irrigation interval is beginning after 20 days from planting

(A) Group I

- Irrigation interval (every 7 days tap water).
- Irrigation interval every 7 days + AsA
- Irrigation interval every 7 days + GSH
- Irrigation interval every 7 days + Pro
- Irrigation interval every 7 days + combined in sequence (AsA+ GSH+ Pro)

(B) Group II

- Irrigation interval (every 14 days tap water).
- Irrigation interval every 14 days + AsA
- Irrigation interval every 14 days + GSH
- Irrigation interval every 14 days + Pro
- Irrigation interval every 14 days + combined in sequence (AsA+ GSH+ Pro)

(C) Group III

- Irrigation interval (every 28 days tap water).
- Irrigation interval every 28 days + AsA
- Irrigation interval every 28 days + GSH
- Irrigation interval every 28 days + Pro
- Irrigation interval every 28 days + combined (AsA+ GSH+ Pro)

The experiment was performed in November 2017 in a greenhouse under the natural conditions (12–14 h photoperiod, 25–27 °C air temperature and 65–70% relative humidity).After 60 days of sowing, ten randomized samples were taken for determination of morphological characteristics, and five plant shoots were used to determine some biochemical measurements and at 150 days the end of the crop cycle to determine specific yield characteristics (ten randomized samples).

#### 3.2.1. Estimation of Photosynthetic Pigments

0.5 g fresh leaves were ground with pestle mortar in liquid nitrogen. The samples were treated with acetone (80%) and the mixture was centrifuged at 20 °C for 5 min at 4500 rpm. To estimate chlorophyll-a chlorophyll-b [72] and carotenoids [73], the filtrate was measured at wavelengths of 470, 652, 665 and 750 nm using a spectrophotometer.

#### 3.2.2. Free Proline Content

0.1 g of dry shoots were ground in 3% sulfosalicylic acid, heated in a water bath (100 °C) for 10 minutes and centrifuged for 15 min at 10,000× *g* after cooling. Proline content was estimated in the resulted supernatant using ninhydrin reagent and read at 520 nm using spectrophotometer [74].

#### 3.2.3. Determination of Ascorbic acid (AsA)

The ascorbic acid content in shoots of chickpea plants was extracted in 6% trichloroacetic acid (TCA) and centrifuged at 1000× *g* for 20 min. Supernatant was measured at wavelength 530 nm using U-Vis spectrophotometer by using the dinitrophenyl hydrazine reagent method [75].

#### 3.2.4. Determination Reduced Glutathione (GSH)

Glutathione was measured in the supernatant of fresh shoots of plants, which extracted in ice-cold 5% (*w*/*v*) sulfosalicylic acid solution and centrifugation at 10,000× *g* for 20 min by using Ellman’s reagent [76]

#### 3.2.5. Determination of Total Soluble Protein

Soluble protein content in fresh shoots of chickpea plants was measured in the supernatant which extracted by cold phosphate buffer (0.05 M at pH 6.5) after centrifugation at 10,000× *g* for 10 min using Folin phenol reagent and measured at 700 nm using a spectrophotometer as described by Lowery et al. [77] technique. The standard is the use of crystalline bovine serum albumin.

#### 3.2.6. Enzyme Extracts Preparation

Using liquid nitrogen, the Fresh shoots (1.0 g) were extracted by in 50 mM phosphate buffer (pH 7.8) for 20 minutes, the homogenate was centrifuged at 15,000 rpm, and the supernatant was used for the following assay of enzyme activity.

#### 3.2.7. Determination of Antioxidant Enzymes

Catalase activity (CAT; EC 1.11.1.6) was determined by the consumption of H_2_O_2_ [78]. Consumption of H_2_O_2_ was monitored spectrophotometrically at 240 nm (e = 39.4 mM^–1^ cm^–1^) for 3 min. Superoxide dismutase activity (SOD; EC 1.15.1.1) was assayed by measuring its ability to inhibit the photochemical reduction of Nitro blue tetrazolium (NBT) [79]. Peroxidase activity (POX: EC 1.11.1.7) was assayed using guaiacol [80] with some modifications. Ascorbate peroxidase activity (APX, EC 1.11.1.11) was determined spectrophotometrically by a decrease in the absorbance at 265 nm (e = 13.7 mM^–1^ cm^–1^) [81]. Glutathione reductase (GR: EC 1.6.4.2) activity was assayed after oxidation of NADPH at 340 nm (extinction coefficient 6.2 mM cm^−1^) for 1 min [82].

### 3.3. Statistical Analysis

The experimental design was completely randomized, so the statistical analyses were conducted using Statistical software SPSS (Social Science version 25.00) at a probability level of 0.05 [83]. Quantitative results with parametric distribution of Levene’s test were conducted using Two-way ANOVA with Tukey Test variance analysis. The confidence interval was set at 95% and the accepted error margin was set at 5%. Graphs were done by GraphPad Prism 8. Discriminant analysis was estimated to show the relationship between quantitative parameters statistical.

## 4. Conclusions

**Figure 5:**
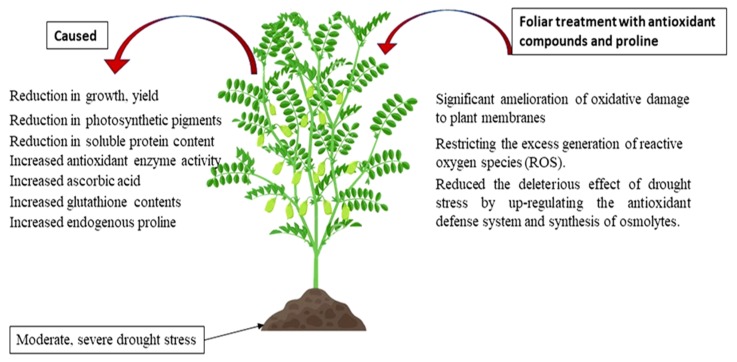
Model showing the regulation of water stress tolerance and application of antioxidants and proline.

Drought stress has a negative effect on plant growth, chlorophyll synthesis and protein content. The deleterious effect of drought stress in plants treated with antioxidants and proline has been improved the growth by positively affected the synthesis of chlorophyll, proline content and the upregulation of antioxidant enzymes. The synergistic effects of the combined antioxidants and proline caused up-regulation of enzymatic and non-enzymatic antioxidant and synthesis of proline in stressed plants which could help in the protection of plants under drought and can be a useful strategy for alleviate the negative impact of drought stress on plant growth (Figure 5).Proline was considered the most effective treatments after combination treatment because proline play a dual function as an osmolyte compound and as an antioxidant. Due to this dual function was superior when used alone compared to the other compounds used.

## Figures and Tables

**Table 1:** Effects of ascorbic acid, glutathione and proline on morphological characteristics of chickpea plants under different irrigation intervals.

Different Levels of Irrigation Interval		Treatments	Shoot Length (cm)	Root Length (cm)	Shoot Fresh Weight FW (g)	Shoot Dry Weight DW (g)	Root Fresh Weight FW (g)	Root Fresh Weight DW (g)
	7 d	0	20.29 ± 1.8 ^d^	9.32 ± 1.11 ^cd^	5.10 ± 0.32 ^e^	2.36 ± 0.22 ^cd^	3.91 ± 0.29 ^ab^	1.4 ± 0.19 ^d^
		AsA	27.64 ± 1.21 ^bc^	12.58 ± 1.61 ^b^	7.88 ± 0.45 ^c^	2.99 ± 0.07 ^bc^	4.98 ± 0.32 ^ab^	1.75 ± 0.1 ^bc^
Control		GSH	29.29 ± 1.19 ^ab^	12.52 ± 1.2 ^b^	8.00 ± 0.62 ^c^	3.25 ± 0.31 ^bc^	4.83 ± 0.53 ^b^	2.07 ± 0.24 ^b^
		Pro	28.43 ± 1.87 ^abc^	13.00 ± 1.72 ^b^	7.23 ± 0.33 ^c^	3.11 ± 0.23 ^bc^	5.10 ± 0.48 ^a^	2.03 ± 0.22 ^b^
		Co	31.33 ± 1.84 ^a^	17.00 ± 1.61 ^a^	10.59 ± 0.64^a^	6.33 ± 0.33 ^a^	5.60 ± 0.47 ^a^	2.59 ± 0.15 ^a^
Water irrigation	14 d	0	12.87 ± 1.03 ^ef^	5.16 ± 1.14 ^f^	3.75 ± 0.8 ^g^	1.38 ± 0.04 ^e^	2.88 ± 0.37 ^e^	0.82 ± 0.26 ^f^
		AsA	18.58 ± 1.72 ^d^	8.32 ± 1.51 ^de^	6.55 ± 0.43 ^d^	2.09 ± 0.3 ^cd^	3.76 ± 0.54 ^d^	1.4 ± 0.28 ^d^
		GSH	18.67 ± 1.56 ^d^	8.67 ± 1.72 ^de^	6.88 ± 0.23 ^d^	2.09 ± 0.25 ^cd^	3.82 ± 0.39 ^d^	1.28 ± 0.2 ^e^
		Pro	19.47 ± 1.56 ^d^	8.44 ± 1.21 ^de^	6.43 ± 0.56 ^d^	2.02 ± 0.27 ^cd^	3.89 ± 0.46 ^d^	1.27 ± 0.25 ^e^
		Co	25 ± 1.32 ^c^	12.00 ± 1.66 ^bc^	9.18 ± 0.29 ^b^	3.97 ± 0.55 ^b^	4.10 ± 0.51 ^c^	1.63 ± 0.27 ^c^
	28 d	0	9.25 ± 2.1 ^f^	3.35 ± 1.82 ^g^	2.21 ± 0.49 ^h^	0.86 ± 0.34 ^f^	1.95 ± 0.64 ^g^	0.63 ± 0.16 ^f^
		AsA	13.25 ± 1.36 ^e^	6.58 ± 1.56 ^def^	4.46 ± 0.24^fg^	1.57 ± 0.07 ^d^	2.88 ± 0.51 ^e^	1.07 ± 0.18 ^g^
		GSH	14.54 ± 1.33 ^e^	5.96 ± 1.27 ^ef^	4.77 ± 0.77 ^f^	1.57 ± 0.05 ^d^	2.92 ± 0.4 ^e^	1.00 ± 0.07 ^g^
		Pro	14.12 ± 1.66 ^e^	6.00 ± 1.51 ^ef^	4.89 ± 0.62 ^f^	1.58 ± 0.24 ^d^	2.96 ± 0.43 ^e^	0.96 ± 0.05 ^g^
		Co	20 ± 1.29 ^d^	8.61 ± 1.23 ^de^	6.31 ± 0.54^de^	2.3 ± 0.22 ^cd^	3.23 ± 0.26 ^f^	1.18 ± 0.1 ^dg^
Significant			**	**	**	**	**	**
Treatments (T)			**	**	**	**	**	**
Water irrigation (WI)			**	**	**	**	*	**
Interaction (T × WI)			ns	ns	ns	*	ns	ns

* Values are means ± SE (*n* = 10). Mean values in each column with the same letters were not significantly different at *p* ≤ 0.05. a, b, c, d, e, f, g, h: Different letters next to the mean values in each column indicate significant difference (LSD *p* ≤ 0.05). * and ** indicate different at *p* value <0.05, 0.01 probability level respectively, “ns” indicate non-significant.

**Table 2:** Effect of ascorbic acid, glutathione and proline on yield attributes of chickpea plants under different levels of irrigation interval.

Different Levels of Irrigation Interval		Treatments	No of Pods Plant^−1^	Pods Weight (g Plant^−1^)	No of Seeds Plant^−1^	100-Seed Weight (g)
	7 d	0	6 ± 0.35 ^e^	1.43 ± 0.04 ^f^	5.33 ± 0.03 ^d^	15.75 ± 0.25 ^d^
		AsA	8 ± 0.03 ^c^	1.90 ± 0.03 ^bc^	7.33 ± 0.04 ^b^	18.98 ± 0.51 ^b^
Control		GSH	8 ± 0.03 ^c^	1.95 ± 0.03 ^bc^	7.67 ± 0.06 ^b^	19.87 ± 0.32 ^b^
		Pro	9 ± 0.03 ^b^	1.97 ± 0.03 ^bc^	7.33 ± 0.06 ^b^	19.45 ± 0.57 ^b^
		Co	11 ± 0.05 ^a^	2.20 ± 0.06 ^a^	9.33 ± 0.06 ^a^	22.58 ± 0.75 ^a^
Water irrigation	14 d	0	3 ± 0.34 ^g^	1.10 ± 0.03 ^g^	3.33 ± 0.03 ^e^	11.34 ± 0.33 ^f^
		AsA	5 ± 0.04 ^f^	1.65 ± 0.04 ^de^	5.33 ± 0.04 ^d^	15.34 ± 0.48 ^e^
		GSH	6 ± 0.06 ^e^	1.77 ± 0.06 ^cd^	5.67 ± 0.06 ^d^	15.98 ± 0.22 ^e^
		Pro	5 ± 0.06 ^f^	1.64 ± 0.06 ^de^	6.33 ± 0.06 ^c^	15.76 ± 0.46 ^e^
		Co	7 ± 0.03 ^d^	1.98 ± 0.03 ^b^	7.33 ± 0.04 ^b^	17.65 ± 0.33 ^c^
	28 d	0	2 ± 0.06 ^h^	0.78 ± 0.06^h^	1.67 ± 0.06 ^f^	9.01 ± 0.01 ^h^
		AsA	4 ± 0.03 ^g^	1.43 ± 0.03 ^f^	4.33 ± 0.06 ^e^	11.12 ± 0.36 ^f^
		GSH	6 ± 0.04 ^e^	1.54 ± 0.04 ^ef^	4.67 ± 0.03 ^e^	11.5 ± 0.42 ^f^
		Pro	5 ± 0.03 ^f^	1.65 ± 0.03 ^de^	5.33 ± 0.02 ^d^	11.3 ± 0.37 ^f^
		Co	7 ± 0.03 ^d^	1.94 ± 0.02 ^bc^	6.67 ± 0.02 ^c^	13.73 ± 0.26 ^d^
Significant			**	**	**	**
Treatments (T)			**	**	**	**
Water irrigation (WI)			**	**	**	**
Interaction (T × WI)			**	ns	ns	ns

* Values are means ± SE (*n* = 10). Mean values in each column with the same letters were not significantly different at *p* ≤ 0.05. a, b, c, d, e, f, g, h: Different letters next to the mean values in each column indicate significant difference (LSD *p* ≤ 0.05). * and ** indicate different at *p* value <0.05, 0.01 probability level respectively, “ns” indicate non-significant.

**Table 3:** Chemical properties of soil.

Total Suspended Solids (TSS) ppm	pH	Electrical Conductivity (EC) mm hos/cm	Cations meq/L				Anions meq/L			
762	7.2	1.19	Na^+^	K^+^	Ca^2+^	Mg^2+^	Cl^−^	SO_4_^−^	HCO_3_^−^	CO_3_^−^
			2.37	0.57	2.22	1	4.4	0.88	1	Zero
